# Identification of Feline Foamy Virus-derived MicroRNAs

**DOI:** 10.1264/jsme2.ME21055

**Published:** 2021-11-13

**Authors:** Shiro Aso, Koichi Kitao, Akira Hashimoto-Gotoh, Shoichi Sakaguchi, Takayuki Miyazawa

**Affiliations:** 1 Laboratory of Virus-Host Coevolution, Institute for Frontier Life and Medical Sciences, Kyoto University, Sakyo-ku, Kyoto 606–8507, Japan; 2 Department of Microbiology and Infection Control, Osaka Medical College, Takatsuki, Osaka 569–8686, Japan

**Keywords:** feline foamy virus, foamy virus, microRNA, spumavirus

## Abstract

MicroRNAs (miRNAs) classified as non-coding RNAs regulate various metabolic systems and viral life cycles. To date, numerous DNA viruses, many of which are members of the herpesvirus family, and a relatively small number of RNA viruses, including retroviruses, have been reported to encode and express miRNAs in infected cells. A few retroviruses have been shown to express miRNAs, and foamy viruses (FVs) were initially predicted by computational analyses to possess miRNA-coding regions. Subsequent studies on simian and bovine FVs confirmed the presence of functional and biologically active miRNA expression cassettes. We herein identified feline FV-derived miRNAs using a small RNA deep sequencing ana­lysis. We confirmed their repressive functions on gene expression by dual-luciferase reporter assays. We found that the seed sequences of the miRNAs identified in the present study were conserved among all previously reported FFV isolates. These results suggest that FFV-derived miRNAs play a pivotal role in FFV infection.

Foamy viruses (FVs), which belong to the subfamily *Spumaretrovirinae* of the family *Retroviridae*, do not cause tumors or other diseases, in contrast to other retroviruses in the subfamily *Orthoretrovirinae* (Falcone *et al.*, 2003; Khan *et al.*, 2018). The FV genome contains 2 long terminal repeats (LTRs) on the 5′ and 3′ ends and encodes 3 canonical proteins of retroviruses, a group-specific antigen (Gag), a polymerase (Pol), envelope (Env) proteins, the accessory protein, Bet, and the regulatory protein, Tas (Lindemann *et al.*, 2021). FVs infect primates (excluding humans), cattle, horses, domestic cats, and wild felids, and have undergone stable FV-host co-speciation for millions of years (Katzourakis *et al.*, 2014). In simian FVs (SFVs), robust and substantial similarities in branching patterns have been demonstrated between hosts and viral phylogenetic trees (Switzer *et al.*, 2005; Ghersi *et al.*, 2015).

MicroRNAs (miRNAs) are defined as non-coding RNAs. They bind to the 3′ untranslated regions (UTRs) of target mRNAs complementarily and induce their cleavage or repress their translation (Bartel, 2018). After being transcribed by RNA pol II or RNA pol III (Ha and Kim, 2014), primary (pri)-miRNAs are processed into precursor (pre)-miRNAs by multiple pathways, such as the Drosha/DiGeorge syndrome chromosomal region (DGCR) pathway (Denli *et al.*, 2004; Gregory *et al.*, 2004). Pre-miRNAs are then cleaved by the Dicer complex to become mature miRNAs and bind to target mRNAs with the RNA-induced silencing complex (RISC) (Han *et al.*, 2004; Gregory *et al.*, 2005). Cellular miRNAs have been reported to regulate various physiological processes, including cell fate, antiviral defense, morphogenesis, and immune responses (Kabekkodu *et al.*, 2018).

Following the discovery of miRNAs expressed by the Epstein-Barr virus (Pfeffer *et al.*, 2004), many DNA viruses, such as herpesviruses and polyomaviruses, have been shown to encode miRNAs (Kincaid and Sullivan, 2012). Although less frequently reported than DNA viruses, virus-derived miRNAs have also been identified in RNA viruses, including retroviruses (Zhan *et al.*, 2020). Kincaid *et al.* (2012) conducted computational analyses to predict the RNA pol III-driven transcription of miRNAs. They found that bovine leukemia virus (BLV) and several FVs possess RNA pol III-transcribed miRNA-like sequences (Kincaid *et al.*, 2012). Small RNA deep sequencing analyses revealed that BLV (Kincaid *et al.*, 2012), bovine FV (BFV) (Whisnant *et al.*, 2014), SFVcae derived from African green monkeys (*Chlorocebus aethiops*) (Kincaid *et al.*, 2014), and SFVmfu derived from Japanese macaques (*Macaca fuscata*) (Hashimoto-Gotoh *et al.*, 2020) express miRNAs. In SFVcae, SFVmfu, and BFV, virus-derived miRNAs have been shown to repress the genes involved in the interferon response, solid tumors, and pro-inflammatory signaling, respectively (Kincaid *et al.*, 2014; Cao *et al.*, 2020; Hashimoto-Gotoh *et al.*, 2020).

The miRNAs derived from BFV (Whisnant *et al.*, 2014), SFVcae (Kincaid *et al.*, 2014), and SFVmfu (Hashimoto-Gotoh *et al.*, 2020) are encoded in the LTR U3 region, and some pri-miRNAs form a unique dumbbell-shaped structure consisting of 2 pre-miRNAs that are adjacent to each other and flanked by few bases. Feline FVs (FFVs) and equine FVs (EFVs) are also predicted to express miRNAs that form dumbbell structures (Materniak-Kornas *et al.*, 2019). However, the expression of FV-derived miRNAs has not been validated using cells infected with these FVs. Therefore, differences in the expression level of each FV-derived miRNA and their functional activities remain unknown.

In the present study, we performed the small RNA deep sequencing of a domestic cat cell line, termed Crandell-Rees feline kidney (CRFK) cells, infected with FFV isolate 159 and identified 8 mature miRNAs. Dual-luciferase reporter assays revealed that these FV-derived miRNAs exhibited repressive activities on the target reporter genes.

## Materials and Methods

### Cell cultures

CRFK cells (ATCC CCL-94) (Crandell *et al.*, 1973) were grown in Dulbecco’s modified Eagle’s medium (DMEM) (Sigma-Aldrich) supplemented with 10% heat-inactivated fetal calf serum (FCS), penicillin (10,000 units mL^–1^), and streptomycin (10,000‍ ‍μg mL^–1^) (Nacalai Tesque) at 37°C in a humidified atmosphere of 5% CO_2_ in the air. Cells were purchased from ATCC and routinely monitored for mycoplasma contamination using the Plasmo Test™ (InvivoGen). The GFP-based FFV-infection indicator cell line, FFG, previously reported (Phung *et al.*, 2003), was grown under the same conditions as CRFK cells.

### Isolation of FFV

To isolate FFV from the urine samples of domestic cats, 500‍ ‍μL of urine samples were centrifuged at 3,000 r.p.m. (2,320×*g*) for 5‍ ‍min to remove debris and then filtered through 450-nm disc filters (PALL). *L*-1-Tosylamide-2-phenylethyl chloromethyl ketone-treated trypsin (Sigma-Aldrich) was added to the samples at a final concentration of 0.1‍ ‍μg mL^–1^, and samples were incubated at 37°C for 15‍ ‍min. The mixture was inoculated into CRFK cells grown in 25-cm^2^ flasks in serum-free DMEM (Sigma-Aldrich) supplemented with antibiotics. After 16 h, inocula were replaced with DMEM supplemented with 2% heat-inactivated FCS and antibiotics (see above). Cultures were incubated at 37°C in a humidified atmosphere with 5% CO_2_ in the air and observed daily for cytopathic effects (CPEs) by light microscopy to confirm virus isolation (Fig. S1). Cultures were passaged by a partial medium change every 3 or 4 days. Surviving cells grow efficiently, and we designated cells persistently infected with FFV as CRFK/FFV(PI) cells. Using PCR, we confirmed that FFVs were present in the genome as proviruses in these cells. To verify that the agent causing CPEs (Sakaguchi *et al.*, 2014) was FFV, we conducted an LTR reporter assay using the culture supernatants of inoculated cells. Briefly, a GFP-based FFV-infection indicator cell line (termed FFG cells) (Phung *et al.*, 2003) was grown in the same media as CRFK cells and inoculated with the culture supernatants of sample-inoculated CRFK cells or naïve CRFK cells as a negative control in the presence of 8‍ ‍μg mL^–1^ of polybrene (hexadimethrine bromide) (Sigma-Aldrich) for viral adsorption. After 2 days, cells were passaged (10-fold dilution) and GFP fluorescence in inoculated cells was observed after 2 days. GFP fluorescence was observed in FFG cells inoculated with the supernatant of FFV-infected cells showing CPE, but not in FFG cells inoculated with the supernatant of naïve CRFK (data not shown). We excluded contamination by other viral agents, including feline morbillivirus and exogenous feline leukemia viruses, by reverse transcription-PCR and PCR, respectively (data not shown) (Miyazawa and Jarrett, 1997). We designated the FFV as isolate 159. Upon infection, FFV isolate 159 induced severe CPEs, which caused large syncytia in CRFK cells (Fig. S1).

### Sequencing and phylogenetic analyses

Genomic DNA was extracted from CRFK/FFV(PI) cells using the DNeasy Blood & Tissue kit (Qiagen). The viral genome was sequenced using PCR-based Sanger sequencing by a commercial DNA sequencing company (FASMAC). The sequence of this FFV has been deposited in GenBank as FFV isolate 159 (GenBank accession number: MW389244). The phylogenetic relationship was inferred by the maximum likelihood method using RAxML v7.2.8 (Stamatakis, 2006). To infer best-fit substitution models, we selected the FLU amino acid replacement model (Nickle *et al.*, 2007) to Env and the HIVb amino acid replacement model (Dang *et al.*, 2010) to the Pol of FFV isolates, which showed the lowest Bayesian information criterion score by ProtTest 3 (Darriba *et al.*, 2011), with gamma-distributed rate heterogeneity and an estimated proportion of invariable sites.

### Construction of plasmids

Genomic DNAs were extracted from uninfected CRFK cells and CRFK/FFV(PI) cells using the QIAamp DNA Blood Mini kit (Qiagen). Genomic DNAs were used as templates to construct miRNA expression plasmids. To clone miRNA expression sites, primers were designed for sequences tens to hundreds of bases upstream/downstream of miRNAs, including the predicted regulatory sequences of miRNAs (Fig. 4A [center and bottom]). All primers used in the present study are listed in Table S1. PCR was performed using KOD-Plus-Neo (TOYOBO) according to the manufacturer’s instructions. In PCR, we used 200-μL thin-walled tubes and a C1000 thermal cycler (BioRad Laboratories). The amplified fragments for FFV-mir-F1 and FFV-mir-F3 were cloned into pUC18 digested with *Hin*dIII/*Bam*HI to become pUC18/FFV-mir-F1 and pUC18/FFV-mir-F3, respectively (Fig. 4A [center and bottom]).

A series of firefly luciferase reporter plasmids were constructed with inserts at the 3′-end of firefly luciferase. Briefly, the human cytomegalovirus (hCMV) enhancer/promoter was cloned into the *Nhe*I/*Hin*dIII sites of pGL3-basic (Promega) to become pGL3-hCMV (Fig. 4A [top]). The duplicated complementary sequence of each mature miRNA (FFV-miR-F1-3p or FFV-miR-F3-3p) was prepared by annealing and extending the synthetic oligos and then cloned into the *Xba*I site of pGL3-hCMV using NEBuilder HiFi DNA Assembly (New England Biolab) for the construction of pGL3-hCMV/ FFV-miR-F1-3p or pGL3-hCMV/ FFV-miR-F3-3p (Fig. 4A [top]).

### Small RNA sequencing ana­lysis

Small RNA sequencing and transcriptome analyses were performed on CRFK and CRFK/FFV(PI) cells. Total RNAs were extracted from cells using RNAzol (Promega), followed by a DNase I (Roche) treatment without a heat inactivation step, and re-extracted using RNAzol. Re-extracted RNAs were further processed by a commercial next-generation sequencing company (Novogene). Sequencing libraries were constructed from total RNAs using the NEBNext Multiplex Small RNA Library Prep Set for Illumina (New England Biolab), which generates strand-specific small RNA libraries. Size selection (18 to 40 nt) of the cDNA libraries was performed using native polyacrylamide gel (12%)-electrophoresis to isolate a fraction containing miRNA-derived cDNAs. Single-end 50-bp sequencing was then performed on an Illumina Hiseq 2500 platform. Sequencing adapter sequences were trimmed from the pre-processed reads using Trimmomatic (ver. 0.38) followed by Fastp (ver. 0.19.4) (Bolger *et al.*, 2014; Chen *et al.*, 2018). Reads were then mapped to the viral genome of FFV isolate 159 (GenBank accession number: MW389244) using Bowtie2 (ver. 2.3.4.3) (Langmead and Salzberg, 2012) and converted to the bam file using Samtools (ver. 1.9) (Li *et al.*, 2009). Bam files were converted to bed files, and the coverage depth was calculated using Bedtools (ver. 2.28.0) (Quinlan and Hall, 2010). The secondary structures of miRNAs were predicted using RNAfold with default parameters (Gruber *et al.*, 2008; Lorenz *et al.*, 2011). Coverage depth was visualized using Microsoft Excel (ver. 16.28). Gained reads were also processed by miRDeep2 to count the number of reads assigned by host-derived miRNAs using 271 feline miRNA precursors and 475 mature sequences identified in a previous feline miRNAome study (Laganà *et al.*, 2017). miRDeep2 uses other tools, including the short read aligner Bowtie and the RNA secondary structure prediction tool RNAfold from the Vienna RNA package 2.0 (Lorenz *et al.*, 2011; Langmead and Salzberg, 2012).

### Dual-luciferase reporter assay

Dual-luciferase reporter assays were conducted to verify that miRNA expression plasmids produce the intended miRNAs. CRFK cells grown in collagen-coated 24-well plates (Iwaki) were co-transfected with 40‍ ‍ng of each firefly luciferase reporter plasmid, 4‍ ‍ng of pRL-TK (Renilla luciferase reporter plasmid) (Promega), and 456‍ ‍ng of each expression plasmid, as indicated in Fig. 4A, using Avalanche®-Everyday Transfection Reagent (EZ Biosystems) according to the manufacturer’s instructions. Cells were harvested 48 h after transfection and subjected to the luciferase assay with the Dual-Glo Luciferase Assay System (Promega) using Lumat LB9507 (Berthold). The significance of differences was assessed using the Student’s *t*-test.

### Accession numbers

Illumina Hiseq sequencing data and nucleotide sequences identified in the present study were deposited to the database of the DNA Data Bank of Japan (DDBJ) with the accession number DRA011435. The nucleotide sequence data of FFV isolate 159 obtained in the present study has been deposited in Genbank with the accession number MW389244.

## Results

### Isolation of FFV isolate 159 from a cat urine sample

**Fig. 1.**
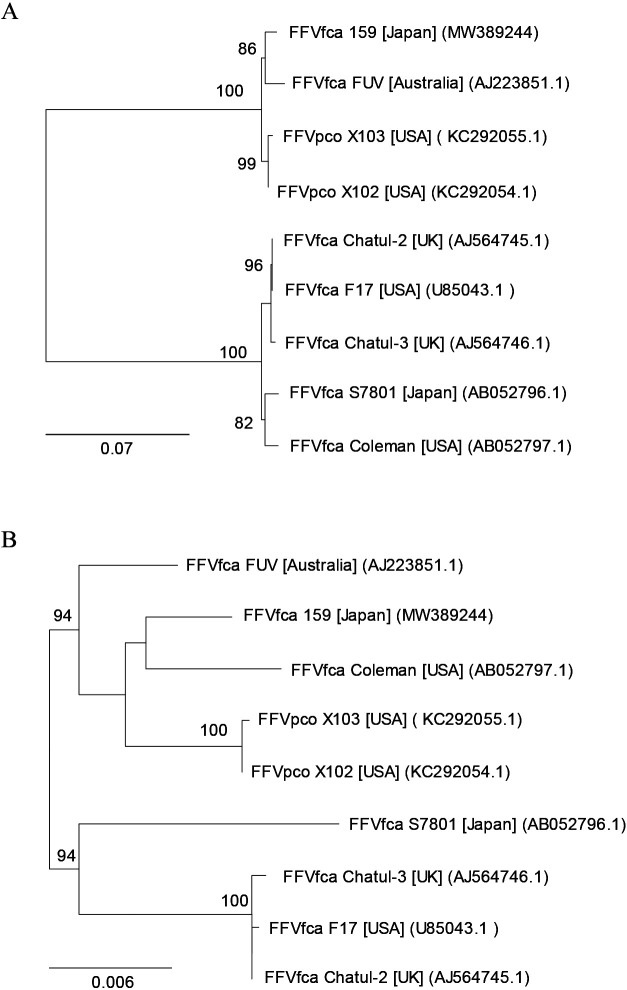
Phylogenetic trees of FFVs. Phylogenetic tree elucidated from the amino acid sequences of (**A**) the Env SU region and (**B**) the Pol of FFVs. Only values >80% for 1,000 fast bootstrapping tests are shown at the nodes. Bar; 0.07 and 0.006 substitutions per amino acid position for (A) and (B), respectively. FFVfca isolates and FFVpco isolates were from domestic cats (*Felis catus*) and pumas, respectively. Square brackets and parentheses indicate the locations of virus isolation and accession numbers, respectively.

We initially attempted to isolate viral agents from cat urine samples using CRFK cells. We successfully isolated feline morbilliviruses and reported them elsewhere (Sakaguchi *et al.*, 2014). We unexpectedly isolated a viral agent that induced severe CPEs with large syncytia, which is typical for FFV infections. We confirmed that the agent causing CPEs was FFV by both PCR and LTR reporter assays, and designated this FFV as isolate 159. We elucidated the entire nucleotide sequence of FFV isolate 159. We then conducted phylogenetic analyses of the amino acid sequences of the Env SU region and the Pol of FFVs (Fig. 1A and B). The Env SU phylogenetic ana­lysis showed that FFV isolate 159 belonged to the FUV type (Fig. 1A).

### FFV expresses 4 viral-derived pri-miRNAs

**Fig. 2.**
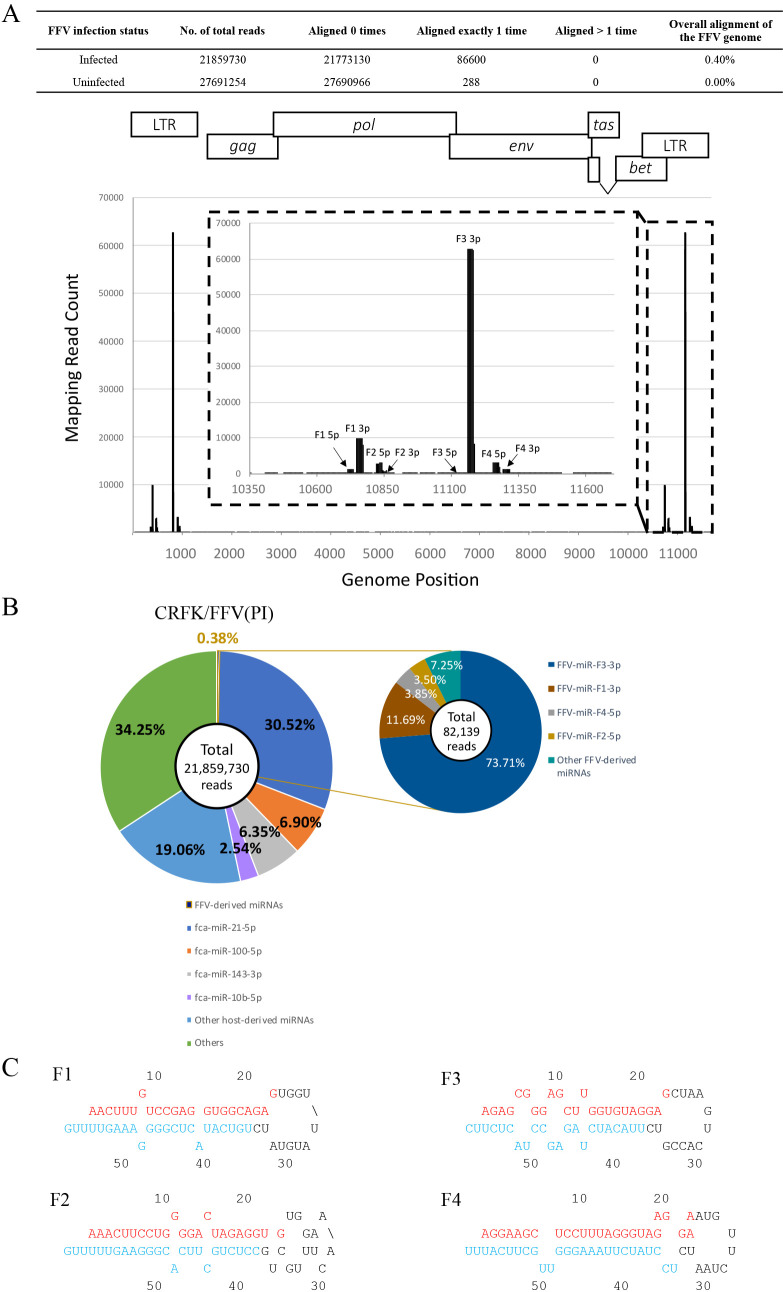
Mapping of FFV-derived miRNAs. (**A**) Mapping of small RNAs in CRFK and CRFK/FFV(PI) cells to FFV genomes. The horizontal axis indicates the genomic position relative to FFV isolate 159 (GenBank accession number: MW389244). The vertical axis shows mapped small RNA read counts. Eight types of mature miRNAs are indicated above the bars. (**B**) Assignment of small RNA reads in CRFK/FFV(PI) cells. The center circle shows the number of total reads obtained by our small RNA sequencing. Auxiliary circles in CRFK/FFV(PI) cells indicate the percentage of reads of FFV-derived mature miRNAs. Others include other small RNAs and RNA degradation products. Other FFV-derived miRNAs indicate the “passenger” strand, which showed a lower expression level than the other functional strand, called the “guide” strand. (**C**) Secondary structures of the 4 identified FFV-derived miRNAs. Red and blue letters indicate 5p and 3p of mature miRNA, respectively.

**Fig. 3.**
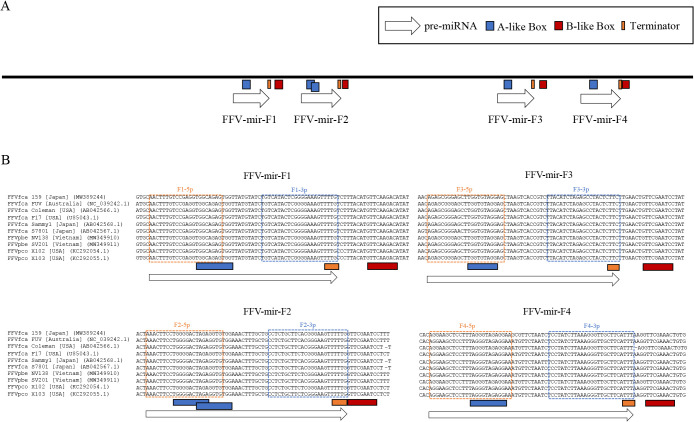
FFV-encoded miRNAs and predicted promoter elements. (A) Schematic of promoter-like elements in the LTR U3 region. (B) Alignments of 5 FFVfca, 2 FFVpco, and 2 FFVpbe isolates available in DDBJ, and FFV isolate 159. Orange and blue boxes show 5p and 3p of mature miRNA, respectively. Gray fill indicates seed6 (positions 2-7 in mature miRNAs).

To identify miRNAs in FFV-infected cells, we performed small RNA sequencing analyses of CRFK cells persistently infected with FFV isolate 159 (CRFK/FFV[PI]). We mapped small RNA reads to FFV isolate 159 and identified 8 mature miRNAs mapping to the U3 region of LTR (Fig. 2A and Table 1). To observe the miRNA expression profile upon FFV infection, we examined the small RNA assignments of CRFK and CRFK/FFV(PI) cells. We calculated the numbers of host-derived miRNA reads using the miRNA sequence data identified in a previous miRNAome ana­lysis (Fig. 2B and Table S2) (Laganà *et al.*, 2017). The results obtained showed that 8 FFV-derived miRNAs contributed to only 0.38% of all small RNA reads in CRFK/FFV(PI) cells (Fig. 2B). The rate of FFV-derived miRNAs in all small reads was slightly different from that of FFV-derived small RNAs in Fig. 2A (0.40%) because aligned small RNA reads in Fig. 2A contained degradation products randomly distributed throughout the FFV genome. On the other hand, previous studies reported that the assignments of virus-derived miRNAs were approximately 32 and 73% for SFVmfu and BFV, respectively (Whisnant *et al.*, 2014; Hashimoto-Gotoh *et al.*, 2020). Consistent with previous studies on other FVs in which miRNAs were derived from RNA pol III, the miRNA cassettes of FFV contained the A/B box sequence, the promoter of RNA pol III (Fig. 3A) (Kassavetis and Geiduschek, 2006; Burke *et al.*, 2014), which predicted that all 4 FFV-derived pri-miRNAs had a single hairpin structure (Fig. 2A, B, C, and Table 1). These results were not consistent with some of the previously identified FV-derived pri-miRNAs, such as SFVcae, SFVmfu, and BFV, which had dumbbell structures (Kincaid *et al.*, 2014; Whisnant *et al.*, 2014; Hashimoto-Gotoh *et al.*, 2020).

We then investigated whether these miRNAs conserved the seed sequences required to recognize target genes among the FFV isolates. miRNA sequences were highly conserved, and all seed sequences (seed6: 2nd–7th base in mature miRNA, which is the most important in seed types [Liu and Wang, 2019]) were identical in 5 FFVfca isolates derived from domestic cats (*Felis catus*), 2 FFVpco isolates derived from pumas (*Puma concolor*), 2 FFVpbe isolates derived from leopard cats (*Prionailurus bengalensis*), available in the NCBI database, and the isolate 159 analyzed in the present study (Fig. 3). These results suggest that the functions of these miRNAs are common among miRNAs derived not only from domestic cats, but also from pumas and leopard cats.

### FFV-derived miRNAs repress gene expression

**Fig. 4.**
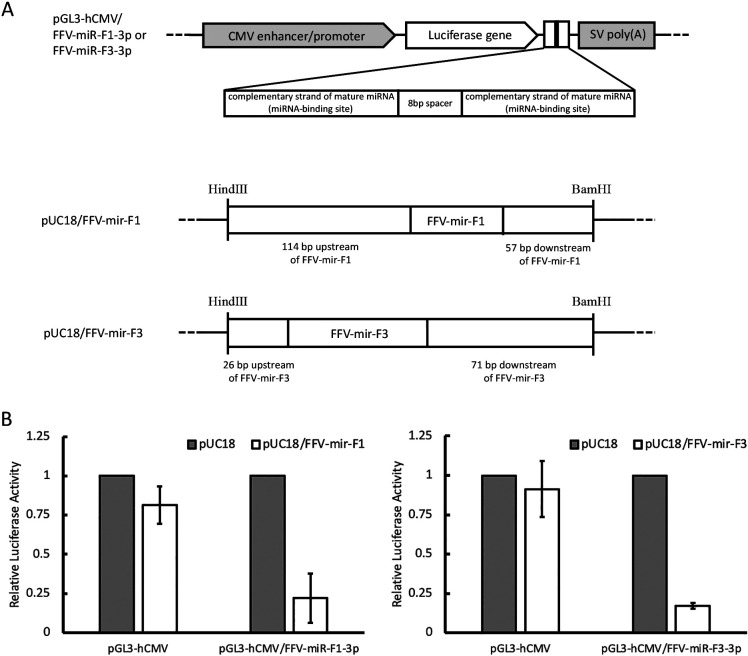
Dual-luciferase reporter assays for miRNA expression plasmids. (**A**) Reporter plasmids were constructed for FFV-miR-F1-3p and FFV-miR-F3-3p (top), and expression plasmids were constructed for FFV-mir-F1 and FFV-mir-F3 (center and bottom). The backbone plasmid (pUC18) for FFV-mir-F1 and FFV-mir-F3 does not contain mammalian promoters; however, the miRNA sequence contains the internal promoter elements (A/B-box sequences). The reporter plasmids contain complementary sequences of mature miRNAs between the firefly luciferase gene and poly(A) signal of the pGL3 vector inserted with the hCMV enhancer/promoter. (**B**) Dual-luciferase reporter assays in CRFK cells using the expression plasmids of FFV-mir-F1 (left panel) and FFV-mir-F3 (right panel). The averages of 3 independent experiments are indicated with standard deviations.

The expression of FFV-miR-F1-3p and FFV-miR-F3-3p was high among the identified miRNAs. We then attempted to conduct functional analyses of these miRNAs. A dual-luciferase reporter assay was performed on CRFK cells to examine whether these miRNAs repress gene expression using pUC18/FFV-mir-F1 and pUC18/FFV-mir-F3, respectively (Fig. 4A). To ensure sensitive detection, we inserted 2 perfectly complementary sequences of mature miRNA into the 3′ UTR of the firefly luciferase gene and used them as reporters (Fig. 4A). As expected, there was an approximately 75% reduction in the expression levels of luciferase genes in both miRNA-expressing cells (Fig. 4B). These results indicate that FFV-miR-F1-3p and FFV-miR-F3-3p have a silencing function for mRNAs possessing complementary sequences in 3′UTR.

## Discussion

In the present study, we isolated FFV isolate 159 from a urine sample. Once FFV infects a cat, it is considered to persist for life (Alke *et al.*, 2000; German *et al.*, 2008). FFV is present in large amounts in saliva and may be isolated from saliva swab samples (Shroyer and Shalaby, 1978). Although there is less free FFV in blood, it may be isolated from buffy coat cells (Shroyer and Shalaby, 1978) and Con-A-stimulated lymphocytes (Miyazawa *et al.*, 1995). FFV has been shown to infect activated lymphocytes and the feline lymphoblastoid cell line MYA-1 (Miyazawa *et al.*, 1995; Ikeda *et al.*, 1997). Leukocytes appear to be persistently infected with FFV in FFV-infected cats. In the present study, in an attempt to isolate feline morbillivirus associated with tubulointerstitial nephritis (Sakaguchi *et al.*, 2014), we isolated a viral agent that causes CPEs typical for FFV in CRFK cells. PCR and RT-PCR analyses showed that the viral agent causing CPE was FFV. According to the literature, FFV has also been isolated from urine (Kruger and Osborne, 1990). Ledesma-Feliciano *et al.* examined the relationship between FFV infection and renal disease in 125 Australian pet cats with and without chronic kidney disease and found no apparent association (Ledesma-Feliciano *et al.*, 2019). In the present study, we sequenced the entire genome of FFV isolate 159. FFV isolates are classified into 2 serotypes, the FUV and F17 types, depending on the Env amino acid sequences (Winkler *et al.*, 1998; Zemba *et al.*, 2000). The phylogenetic ana­lysis of Env SU indicated that this isolate belonged to the FUV type (Fig. 1A).

The expression of 4 types of pri-miRNAs was speculated based on RNA pol III promoter sequences (Fig. 3A). Among them, FFV-miR-F1 and FFV-miR-F3 were predicted in a previous *in silico* ana­lysis (Kincaid *et al.*, 2014) and accounted for more than 90% of the identified FFV-derived miRNAs (Fig. 2B). We demonstrated using dual-luciferase reporter assays that FFV-miR-F1-3p and FFV-miR-F3-3p functioned as miRNAs to repress gene expression against their target sequences (Fig. 4). It is important to note that in comparisons with similar experiments on BFV, SFVcae, and SFVmfu (Whisnant *et al.*, 2014; Kincaid *et al.*, 2014; Hashimoto-Gotoh *et al.*, 2020), FFV miRNAs only achieved moderate suppression. This may be due to miRNA expression in FFV being inhibited by flanking genomic sequences, as was previously shown for BFV (Cao *et al.*, 2018). FFV-miR-F2 and FFV-miR-F4 are novel FFV-derived miRNAs identified in the present study (Fig. 2). All of these FFV-derived pri-miRNAs were predicted to have a single hairpin structure and, thus, did not have the dumbbell structure observed in SFVmfu (Hashimoto-Gotoh *et al.*, 2020), SFVcae (Kincaid *et al.*, 2014), and BFV (Whisnant *et al.*, 2014).

Sequence comparisons of FFV isolate 159 with 9 other FFV isolates, including FFV from pumas and leopard cats, showed that mature miRNA sequences were highly conserved and seed sequences were perfectly conserved (Fig. 3B). In the case of FFV isolates from pumas, in addition to the isolates aligned in Fig. 3B, we added 33 isolates reported in 2020 for sequence comparisons (Kraberger *et al.*, 2020), and the seed sequences of these isolates also showed a complete identity (data not shown). This result suggests that FFV-derived miRNAs have a common function in cats and other feline species. Many interspecies infections by FFV have been reported (Daniels *et al.*, 1999; Phung *et al.*, 2001; Kraberger *et al.*, 2020); therefore, FFV-derived miRNAs may function in other feline species, such as pumas and leopard cats. On the other hand, the seed sequences of SFVmfu and SFVcae-derived miRNAs are not conserved (Kincaid *et al.*, 2014; Hashimoto-Gotoh *et al.*, 2020), suggesting that different miRNAs are involved in infections by SFVs in each primate species.

The target genes of miRNAs are diverse, and target gene prediction using a combination of an RNA deep ana­lysis and cross-linking immunoprecipitation ana­lysis of human-derived miRNAs predicted an average of 90 target genes per miRNA (Liu and Wang, 2019). Therefore, the accumulation of comprehensive knowledge on gene function and accurate prediction tools for miRNA target genes are essential for identifying biologically important target genes of miRNAs. However, due to the lack of prediction tools for miRNA target genes in cats, difficulties are associated with the identification of target genes that are important for FFV infection. Therefore, although we did not search for the target genes of FFV-derived miRNAs in the present study, it is important to predict target genes when reliable target gene prediction tools become available in cats. Since previous studies reported that FV-derived miRNAs, such as SFVcae, SFVmfu, and BFV, suppress genes related to immune responses and tumor suppression in the host, it is possible that FFV-derived miRNAs also act on these metabolic pathways and help FFV infect hosts (Kincaid *et al.*, 2014; Cao *et al.*, 2020; Hashimoto-Gotoh *et al.*, 2020). In addition, an evaluation of infectivity using the miRNA deletion mutants of BFV showed that BFV-derived miRNAs contributed to improved infection efficiency (Cao *et al.*, 2020). Therefore, studies using miRNA deletion mutants will provide novel insights into the biological functions of FFV-derived miRNAs in the future.

## Citation

Aso, S., Kitao, K., Hashimoto-Gotoh, A., Sakaguchi, S., and Miyazawa, T. (2021) Identification of Feline Foamy Virus-derived MicroRNAs. *Microbes Environ ***36**: ME21055.

https://doi.org/10.1264/jsme2.ME21055

## Supplementary Material

Supplementary Material

## Figures and Tables

**Table 1.** List of mature miRNA sequences identified in the present study

Name	Sequence (5′-3′)	Length (nt)	Start position	End position	Number of reads
FFV-miR-F1-5p	AACUUUGUCCGAGGUGGCAGAG	22	10,707	10,728	1,230
FFV-miR-F1-3p	UGUCAUACUCGGGGAAAGUUUUG	23	10,741	10,763	9,951
FFV-miR-F2-5p	AAACUUCCUGGGGACUAGAGGUG	23	10,813	10,835	2,981
FFV-miR-F2-3p	CCUCUGCUUCACGGGAAGUUUUUG	24	10,850	10,873	317
FFV-miR-F3-5p	AGAGCGGGAGCUUGGUGUAGGAG	23	11,118	11,140	242
FFV-miR-F3-3p	UUACAUCUAGAGCCUACUCUUC	22	11,154	1,175	62,743
FFV-miR-F4-5p	AGGAAGCUCCUUUAGGGUAGAGGAA	25	11,248	11,272	3,275
FFV-miR-F4-3p	UCCUAUCUUAAAGGGUUGCUUCAUUU	26	11,284	11,309	1,400
