# Supplementary material for: Identification of Feline Foamy Virus-derived MicroRNAs
Source: Microbes Environ. 2021 Nov 13;36(4):ME21055. doi: 10.1264/jsme2.ME21055 (PMC8674446; doi:10.1264/jsme2.ME21055)
Supplement: Supplementary file 1 — Supplementary Material [file 36_21055_s1.pdf]

Table S1. Primers used in this study

Name	Purpose of PCR	Template	Target sequence	Sequence (5′ - 3′)
FFVmiRNA_F1_F	To construct miRNA expression plasmids	genomic DNA of CRFK cells infected with FFV	pre-miRNA sequence of miRNA FFV F1	CCCAAGCTTCATAGCCTGTGATCCTG
FFVmiRNA_F1_R				CGCGGATCCGGAAGTTTAGTCAGGAA
FFVmiRNA_F3_F			pre-miRNA sequence of miRNA FFV F3	CCCAAGCTTGAGAAGAACAACTTTCG
FFVmiRNA_F3_R				CGCGGATCCTGCACGAGTCATGCTTT
FFV_F1_comp_F	To construct luciferase reporter plasmids	No template (annealing and extending primers)	Complementary sequences of miRNA FFV F1	GATCGCCGTGTAATTCAAAACTTTCCCCGAGTATGACAATCGATCGCAAAAC
FFV_F1_comp_R				CCGGCCGCCCCGACTTGTCATACTCGGGGAAAGTTTTGCGATCGATTGTCAT
FFV_F3_comp_F			Complementary sequences of miRNA FFV F3	GATCGCCGTGTAATTGAAGAGTAGGCTCTAGATGTAAATCGATCGGAAGAGT
FFV_F3_comp_R				CCGGCCGCCCCGACTTTACATCTAGAGCCTACTCTTCCGATCGATTTACATC

Table S2. The read counts of host-derived miRNAs of CRFK (NC) or CRFK/FFV (PI)

miRNA	NC_read_count	PI_read_count	precursor
**fca-miR-21-5p**	14074467	6671652	fca-mir-21
**fca-miR-100-5p**	2806235	1508584	fca-mir-100
**fca-miR-143-3p**	1210515	1388502	fca-mir-143
**fca-miR-10b-5p**	187783	555919	fca-mir-10b
**fca-let-7i-5p**	351476	444677	fca-let-7i
**fca-let-7f-5p**	252117	431679	fca-let-7f
**fca-miR-26a-5p**	268767	425312	fca-mir-26a-2
**fca-miR-26a-5p**	268767	425309	fca-mir-26a-1
**fca-miR-10a-5p**	230773	287699	fca-mir-10a
**fca-miR-532-5p**	119912	252193	fca-mir-532
**fca-miR-27a-3p**	78176	162406	fca-mir-27a
**fca-miR-24-3p**	198337	139890	fca-mir-24-1
**fca-miR-24-3p**	198337	139890	fca-mir-24-2
**fca-miR-99b-5p**	79793	135114	fca-mir-99b
**fca-miR-27b-3p**	50066	124391	fca-mir-27b
**fca-let-7g-5p**	42644	122317	fca-let-7g
**fca-miR-7-5p**	496433	115997	fca-mir-7-1
**fca-miR-7-5p**	494719	115468	fca-mir-7-2
**fca-miR-7-5p**	494547	115435	fca-mir-7-3
**fca-let-7a-5p**	67462	109295	fca-let-7a-2
**fca-miR-99a-5p**	46177	102919	fca-mir-99a
**fca-miR-29a-3p**	33461	100335	fca-mir-29a
**fca-let-7a-5p**	62861	99364	fca-let-7a-1
**fca-miR-101-3p**	43204	94923	fca-mir-101a
**fca-miR-101-3p**	43204	94923	fca-mir-101b
**fca-miR-125b-5p**	91876	90888	fca-mir-125b-1
**fca-miR-125b-5p**	91637	90312	fca-mir-125b-2
**fca-miR-140-3p**	40606	52564	fca-mir-140
**fca-miR-1-3p**	30988	52243	fca-mir-1-1
**fca-miR-1-3p**	30988	52243	fca-mir-1-2
**fca-miR-340-5p**	28024	48273	fca-mir-340
**fca-miR-199a-5p**	37837	45109	fca-mir-199a-1
**fca-miR-199a-5p**	37837	45109	fca-mir-199a-2
**fca-miR-450b-5p**	34574	38574	fca-mir-450b
**fca-miR-191-5p**	25473	32068	fca-mir-191
**fca-miR-152-3p**	23220	30030	fca-mir-152
**fca-miR-378-3p**	12184	28637	fca-mir-378
**fca-miR-103-3p**	11191	27230	fca-mir-103
**fca-miR-16-5p**	14413	24455	fca-mir-16-2
**fca-miR-182**	12180	22718	fca-mir-182
**fca-miR-93-5p**	26843	22178	fca-mir-93
**fca-miR-185-5p**	10788	22062	fca-mir-185
**fca-miR-199-3p**	18286	21956	fca-mir-199a-1
**fca-miR-199-3p**	18286	21956	fca-mir-199a-2
**fca-miR-199-3p**	18286	21956	fca-mir-199b
**fca-miR-196b-5p**	22988	21907	fca-mir-196b
**fca-miR-26b-5p**	10127	21804	fca-mir-26b
**fca-miR-186-5p**	7602	20715	fca-mir-186
**fca-miR-23b-3p**	9544	20057	fca-mir-23b
**fca-miR-16-5p**	11830	19936	fca-mir-16-1
**fca-miR-181a-5p**	3084	19197	fca-mir-181a-1
**fca-miR-30c-5p**	8982	19179	fca-mir-30c-1

**fca-miR-30c-5p**	8982	19179	fca-mir-30c-2
**fca-miR-181a-5p**	3076	19168	fca-mir-181a-2
**fca-let-7e-5p**	7791	18896	fca-let-7e
**fca-miR-183-5p**	10796	17972	fca-mir-183
**fca-miR-27a-5p**	29553	17183	fca-mir-27a
**fca-miR-34c-5p**	1866	15647	fca-mir-34c
**fca-miR-192-5p**	10290	14225	fca-mir-192
**fca-miR-423-5p**	12274	13569	fca-mir-423
**fca-miR-149-5p**	13853	11330	fca-mir-149
**fca-miR-106b-3p**	7442	11063	fca-mir-106b
**fca-miR-34a-5p**	5098	10898	fca-mir-34a
**fca-miR-140-5p**	5592	10593	fca-mir-140
**fca-miR-139-5p**	215	9304	fca-mir-139
**fca-miR-151-3p**	4799	8995	fca-mir-151
**fca-miR-221-5p**	25031	8700	fca-mir-221
**fca-miR-361-3p**	2027	8679	fca-mir-361
**fca-miR-98-5p**	9093	8484	fca-miR-98
**fca-miR-181b-5p**	1841	7988	fca-mir-181b-1
**fca-miR-181b-5p**	1816	7902	fca-mir-181b-2
**fca-miR-146b-5p**	6744	7849	fca-mir-146b
**fca-miR-582-5p**	3823	7773	fca-mir-582
**fca-let-7c-5p**	3960	7662	fca-let-7c
**fca-miR-22-3p**	1734	7150	fca-mir-22
**fca-miR-660-5p**	3100	6772	fca-mir-660
**fca-miR-20a-5p**	11071	6713	fca-mir-20a
**fca-miR-423-3p**	9073	6422	fca-mir-423
**fca-miR-99b-3p**	3469	6040	fca-mir-99b
**fca-miR-499-5p**	2373	5844	fca-mir-499
**fca-miR-374b-5p**	3638	5812	fca-mir-374b
**fca-miR-128-3p**	1840	5467	fca-mir-128-1
**fca-miR-128-3p**	1840	5467	fca-mir-128-2
**fca-miR-2387-5p**	5087	5347	fca-mir-2387
**fca-miR-30b-5p**	7909	5253	fca-mir-30b
**fca-miR-365-3p**	3690	5058	fca-mir-365
**fca-miR-129-5p**	2513	4996	fca-mir-129-2
**fca-miR-129-5p**	2508	4979	fca-mir-129-1
**fca-miR-1271-5p**	4475	4826	fca-mir-1271
**fca-miR-362-5p**	2530	4703	fca-mir-362
**fca-miR-374a-5p**	1693	4690	fca-mir-374a
**fca-miR-106b-5p**	4856	4403	fca-mir-106b
**fca-miR-196a-5p**	20233	4319	fca-mir-196a
**fca-miR-122-5p**	609	4294	fca-mir-122
**fca-miR-25-3p**	7421	4222	fca-mir-25
**fca-miR-6529-5p**	3141	4089	fca-mir-6529
**fca-miR-22-5p**	1812	3817	fca-mir-22
**fca-miR-378-5p**	2517	3751	fca-mir-378
**fca-miR-218-5p**	3933	3626	fca-mir-218-1
**fca-miR-218-5p**	3932	3623	fca-mir-218-2
**fca-miR-19b-3p**	6661	3553	fca-mir-19b
**fca-miR-148b-3p**	1177	3316	fca-mir-148b
**fca-miR-155-5p**	10543	3236	fca-mir-155
**fca-miR-17-5p**	4688	3182	fca-mir-17
**fca-miR-222-3p**	5443	3111	fca-mir-222
**fca-miR-130b-5p**	3569	3039	fca-mir-130b

**fca-miR-125b-1-3p**	5461	2959	fca-mir-125b-1
**fca-miR-503-5p**	2990	2640	fca-mir-503
**fca-miR-1307-3p**	3273	2577	fca-mir-1307
**fca-miR-1343-3p**	1185	2568	fca-mir-1343
**fca-miR-24-2-5p**	2345	2481	fca-mir-24-2
**fca-miR-15b-5p**	1284	2156	fca-mir-15b
**fca-miR-320a-3p**	2117	2098	fca-mir-320a
**fca-miR-502-3p**	1082	2049	fca-mir-502
**fca-miR-133a-3p**	1855	2036	fca-mir-133a-1
**fca-miR-133a-3p**	1855	2036	fca-mir-133a-2
**fca-miR-151-5p**	1420	1986	fca-mir-151
**fca-miR-184-3p**	1928	1835	fca-mir-184
**fca-miR-574-5p**	1761	1800	fca-mir-574
**fca-miR-125b-2-3p**	617	1798	fca-mir-125b-2
**fca-miR-193a-5p**	2473	1780	fca-mir-193a
**fca-miR-148a-3p**	688	1684	fca-mir-148a
**fca-miR-331-5p**	1791	1664	fca-mir-331
**fca-miR-221-3p**	4398	1623	fca-mir-221
**fca-miR-96-5p**	1366	1601	fca-mir-96
**fca-miR-152-5p**	1308	1440	fca-mir-152
**fca-miR-148a-5p**	1351	1349	fca-mir-148a
**fca-miR-143-5p**	869	1299	fca-mir-143
**fca-miR-652-3p**	2112	1250	fca-mir-652
**fca-miR-574-3p**	852	1234	fca-mir-574
**fca-miR-130a-3p**	943	1207	fca-mir-130a
**fca-miR-181d-5p**	161	1182	fca-mir-181d
**fca-miR-185-3p**	372	1135	fca-mir-185
**fca-miR-214-3p**	77	1113	fca-mir-214
**fca-miR-181a-1-3p**	101	1085	fca-mir-181a-1
**fca-miR-361-5p**	407	1059	fca-mir-361
**fca-miR-542-3p**	675	1031	fca-mir-542
**fca-miR-205-5p**	15	942	fca-mir-205
**fca-miR-375-3p**	70	817	fca-mir-375
**fca-miR-424-5p**	530	812	fca-mir-424
**fca-miR-chrC2_22423-3p**	6	790	fca-mir-chrC2_22423
**fca-miR-1306-5p**	1173	767	fca-mir-1306
**fca-miR-18a-5p**	929	754	fca-mir-18a
**fca-miR-130b-3p**	1628	711	fca-mir-130b
**fca-miR-15a-5p**	429	705	fca-mir-15a
**fca-miR-197-3p**	342	693	fca-mir-197
**fca-miR-27b-5p**	905	691	fca-mir-27b
**fca-miR-350-5p**	636	679	fca-mir-350
**fca-miR-193b-5p**	794	642	fca-mir-193b
**fca-miR-340-3p**	469	629	fca-mir-340
**fca-miR-29b-3p**	156	626	fca-mir-29b-2
**fca-miR-194-5p**	355	615	fca-mir-194
**fca-miR-chrC1_18846-5p**	370	603	fca-mir-chrC1_18846
**fca-miR-92a-3p**	1564	603	fca-mir-92a
**fca-miR-199b-5p**	212	588	fca-mir-199b
**fca-miR-181c-3p**	80	585	fca-mir-181c
**fca-miR-486-5p**	175	562	fca-mir-486-2
**fca-miR-486-5p**	179	562	fca-mir-486-1
**fca-miR-195-5p**	103	537	fca-mir-195
**fca-miR-29a-5p**	174	512	fca-mir-29a

**fca-miR-188-5p**	336	512	fca-mir-188
**fca-miR-210-3p**	2301	503	fca-mir-210
**fca-let-7a-1-3p**	618	485	fca-let-7a-1
**fca-miR-532-3p**	379	471	fca-mir-532
**fca-miR-505-3p**	255	461	fca-mir-505
**fca-miR-1249-3p**	382	434	fca-mir-1249
**fca-miR-193b-3p**	1391	434	fca-mir-193b
**fca-miR-3548-5p**	55	424	fca-mir-3548
**fca-miR-chrUn_JH409706_38826-3p**	725	365	fca-mir-chrUn_JH409706_38826
**fca-miR-1296-5p**	352	347	fca-mir-1296
**fca-miR-671-5p**	563	341	fca-mir-671
**fca-miR-100-3p**	614	316	fca-mir-100
**fca-miR-502-5p**	241	307	fca-mir-502
**fca-miR-190a-5p**	199	291	fca-mir-190a
**fca-miR-1307-5p**	364	279	fca-mir-1307
**fca-miR-138-1-5p**	248	272	fca-mir-138-1
**fca-miR-214-5p**	20	262	fca-mir-214
**fca-miR-chrE3_33626-5p**	509	259	fca-mir-chrE3_33626
**fca-miR-138-1-5p**	236	255	fca-mir-138-2
**fca-miR-138-2-5p**	236	255	fca-mir-138-2
**fca-miR-130a-5p**	182	231	fca-mir-130a
**fca-miR-628-5p**	98	221	fca-mir-628
**fca-miR-664-3p**	103	193	fca-mir-664
**fca-miR-652-5p**	391	192	fca-mir-652
**fca-miR-1306-3p**	267	191	fca-mir-1306
**fca-miR-133a-5p**	202	184	fca-mir-133a-1
**fca-miR-326-3p**	149	176	fca-mir-326
**fca-miR-215-5p**	193	176	fca-mir-215
**fca-miR-374a-3p**	40	173	fca-mir-374a
**fca-miR-181a-2-3p**	26	169	fca-mir-181a-2
**fca-miR-30c-1-3p**	53	167	fca-mir-30c-1
**fca-miR-497-5p**	70	159	fca-mir-497
**fca-miR-129-2-3p**	154	159	fca-mir-129-2
**fca-miR-133a-5p**	168	154	fca-mir-133a-2
**fca-miR-128-1-5p**	193	148	fca-mir-128-1
**fca-miR-210-5p**	402	140	fca-mir-210
**fca-miR-181c-5p**	21	139	fca-mir-181c
**fca-miR-483-3p**	202	136	fca-mir-483
**fca-miR-708-3p**	41	128	fca-mir-708
**fca-miR-615-3p**	324	127	fca-mir-615
**fca-miR-24-1-5p**	82	126	fca-mir-24-1
**fca-miR-424-3p**	88	125	fca-mir-424
**fca-let-7a-2-3p**	159	125	fca-let-7a-2
**fca-miR-708-5p**	39	119	fca-mir-708
**fca-miR-328-3p**	66	116	fca-mir-328
**fca-miR-26a-2-3p**	65	110	fca-mir-26a-2
**fca-miR-93-3p**	73	110	fca-mir-93
**fca-miR-350-3p**	30	109	fca-mir-350
**fca-miR-191-3p**	127	108	fca-mir-191
**fca-miR-542-5p**	117	107	fca-mir-542
**fca-miR-29b-2-5p**	56	104	fca-mir-29b-2
**fca-let-7i-3p**	167	104	fca-let-7i
**fca-miR-342-3p**	23	99	fca-mir-342
**fca-miR-15b-3p**	91	87	fca-mir-15b

**fca-miR-181b-1-3p**	23	85	fca-mir-181b-1
**fca-let-7e-3p**	30	82	fca-let-7e
**fca-miR-206-3p**	21	81	fca-mir-206
**fca-miR-3548-3p**	14	80	fca-mir-3548
**fca-miR-21-3p**	132	80	fca-mir-21
**fca-miR-chrE3_33626-3p**	42	79	fca-mir-chrE3_33626
**fca-miR-139-3p**	1	76	fca-mir-139
**fca-miR-30c-2-3p**	25	76	fca-mir-30c-2
**fca-miR-195-3p**	8	73	fca-mir-195
**fca-miR-582-3p**	49	71	fca-mir-582
**fca-miR-10b-3p**	16	70	fca-mir-10b
**fca-miR-33-5p**	39	68	fca-mir-33
**fca-miR-335-5p**	158	64	fca-mir-335
**fca-miR-590-5p**	35	60	fca-mir-590
**fca-miR-17-3p**	109	56	fca-mir-17
**fca-miR-342-5p**	48	55	fca-mir-342
**fca-miR-124-3p**	26	52	fca-mir-124-1
**fca-miR-124-3p**	26	52	fca-mir-124-2
**fca-miR-193a-3p**	448	51	fca-mir-193a
**fca-miR-18a-3p**	104	48	fca-mir-18a
**fca-miR-411-5p**	521	48	fca-mir-411
**fca-miR-chrD4_31051-5p**	8	43	fca-mir-chrD4_31051
**fca-miR-590-3p**	59	42	fca-mir-590
**fca-miR-505-5p**	42	41	fca-mir-505
**fca-miR-10a-3p**	58	40	fca-mir-10a
**fca-miR-200a-5p**	11	38	fca-mir-200a
**fca-miR-660-3p**	31	36	fca-mir-660
**fca-let-7f-3p**	70	36	fca-let-7f
**fca-miR-chrB1_11053-3p**	122	34	fca-mir-chrB1_11053
**fca-miR-335-3p**	275	33	fca-mir-335
**fca-miR-204-5p**	50	32	fca-mir-204
**fca-miR-200b-3p**	3	31	fca-mir-200b
**fca-miR-874-5p**	0	30	fca-mir-874
**fca-miR-33-3p**	32	29	fca-mir-33
**fca-miR-1-1-5p**	8	28	fca-mir-1-1
**fca-miR-30b-3p**	5	27	fca-mir-30b
**fca-miR-chrE1_32174-3p**	8	27	fca-mir-chrE1_32174
**fca-miR-874-3p**	0	26	fca-mir-874
**fca-miR-374b-3p**	9	26	fca-mir-374b
**fca-miR-331-3p**	37	26	fca-mir-331
**fca-miR-34a-3p**	3	24	fca-mir-34a
**fca-let-7g-3p**	60	24	fca-let-7g
**fca-miR-200a-3p**	2	23	fca-mir-200a
**fca-let-7c-3p**	20	23	fca-let-7c
**fca-miR-1301-3p**	14	22	fca-mir-1301
**fca-miR-129-1-3p**	20	22	fca-mir-129-1
**fca-miR-449-5p**	25	22	fca-mir-449
**fca-miR-132-5p**	13	21	fca-mir-132
**fca-miR-23b-5p**	20	20	fca-mir-23b
**fca-miR-26b-3p**	12	19	fca-mir-26b
**fca-miR-chrE2_33458-3p**	20	19	fca-mir-chrE2_33458
**fca-miR-429-3p**	0	17	fca-mir-429
**fca-miR-135a-5p**	3	17	fca-mir-135a-1
**fca-miR-135a-5p**	3	17	fca-mir-135a-2

**fca-miR-6529-3p**	4	17	fca-mir-6529
**fca-miR-491-5p**	17	17	fca-mir-491
**fca-miR-379-5p**	416	17	fca-mir-379
**fca-miR-196b-3p**	2	15	fca-mir-196b
**fca-miR-493-5p**	0	14	fca-mir-493
**fca-miR-3085-5p**	5	14	fca-mir-3085
**fca-miR-362-3p**	9	14	fca-mir-362
**fca-miR-chrB2_13690-3p**	11	14	fca-mir-chrB2_13690
**fca-miR-200c-3p**	18	12	fca-mir-200c
**fca-miR-126-3p**	81	12	fca-mir-126
**fca-miR-491-3p**	5	11	fca-mir-491
**fca-miR-551a-3p**	10	11	fca-mir-551a
**fca-miR-chrB2_13690-5p**	70	11	fca-mir-chrB2_13690
**fca-miR-124-5p**	2	10	fca-mir-124-1
**fca-miR-124-5p**	2	10	fca-mir-124-2
**fca-miR-3085-3p**	4	10	fca-mir-3085
**fca-miR-106a-5p**	19	10	fca-mir-106a
**fca-miR-chrC2_22423-5p**	0	9	fca-mir-chrC2_22423
**fca-miR-181b-2-3p**	8	9	fca-mir-181b-2
**fca-miR-135b-5p**	0	8	fca-mir-135b
**fca-miR-132-3p**	8	8	fca-mir-132
**fca-miR-103-5p**	9	8	fca-mir-103
**fca-miR-chrE3_33972-3p**	19	8	fca-mir-chrE3_33972
**fca-miR-138-1-3p**	9	7	fca-mir-138-1
**fca-miR-188-3p**	0	6	fca-mir-188
**fca-miR-592-5p**	0	6	fca-mir-592
**fca-miR-127-3p**	6	6	fca-mir-127
**fca-miR-chrB1_11053-5p**	12	6	fca-mir-chrB1_11053
**fca-miR-503-3p**	19	6	fca-mir-503
**fca-miR-382-5p**	31	6	fca-mir-382
**fca-miR-101a-5p**	0	5	fca-mir-101a
**fca-miR-127a-5p**	1	5	fca-mir-127
**fca-miR-16-2-3p**	1	5	fca-mir-16-2
**fca-miR-chrE3_34060-5p**	3	5	fca-mir-chrE3_34060
**fca-miR-98-3p**	5	5	fca-miR-98
**fca-miR-202-5p**	5	5	fca-mir-202
**fca-miR-183-3p**	11	5	fca-mir-183
**fca-miR-7a-1-3p**	26	5	fca-mir-7-1
**fca-miR-299a-3p**	36	5	fca-mir-299a
**fca-miR-302d-1-3p**	1	4	fca-mir-302d-1
**fca-miR-514-5p**	1	4	fca-mir-514
**fca-miR-chrC2_23051-3p**	1	4	fca-mir-chrC2_23051
**fca-miR-295-3p**	2	4	fca-mir-295
**fca-miR-219-3p**	3	4	fca-mir-219
**fca-miR-6715a-3p**	4	4	fca-mir-6715a
**fca-miR-chrE3_34145-5p**	6	4	fca-mir-chrE3_34145
**fca-miR-409-3p**	11	4	fca-mir-409
**fca-miR-485-5p**	12	4	fca-mir-485
**fca-miR-182-3p**	0	3	fca-mir-182
**fca-miR-200b-5p**	2	3	fca-mir-200b
**fca-miR-192-3p**	3	3	fca-mir-192
**fca-miR-543-3p**	3	3	fca-mir-543
**fca-miR-153-1-5p**	4	3	fca-mir-153-1
**fca-miR-3959-5p**	10	3	fca-mir-3959

**fca-miR-150-5p**	11	3	fca-mir-150
**fca-miR-218-2-3p**	0	2	fca-mir-218-2
**fca-miR-219-5p**	0	2	fca-mir-219
**fca-miR-383-5p**	0	2	fca-mir-383
**fca-miR-495-3p**	0	2	fca-mir-495
**fca-miR-8908n-3p**	0	2	fca-mir-8908n
**fca-miR-chrX_38640-3p**	0	2	fca-mir-chrX_38640
**fca-miR-26a-1-3p**	2	2	fca-mir-26a-1
**fca-miR-432-5p**	2	2	fca-mir-432
**fca-miR-chrF2_35515-3p**	4	2	fca-mir-chrF2_35515
**fca-miR-628-3p**	5	2	fca-mir-628
**fca-miR-chrE1_31800-3p**	5	2	fca-mir-chrE1_31800
**fca-miR-1271-3p**	13	2	fca-mir-1271
**fca-miR-409-5p**	15	2	fca-mir-409
**fca-miR-122-3p**	0	1	fca-mir-122
**fca-miR-135a-3p**	0	1	fca-mir-135a-1
**fca-miR-144-5p**	0	1	fca-mir-144
**fca-miR-215-3p**	0	1	fca-mir-215
**fca-miR-216a-5p**	0	1	fca-mir-216a
**fca-miR-296-3p**	0	1	fca-mir-296
**fca-miR-323a-3p**	0	1	fca-mir-323a
**fca-miR-376a-5p**	0	1	fca-mir-376a-1
**fca-miR-379-3p**	0	1	fca-mir-379
**fca-miR-381-5p**	0	1	fca-mir-381
**fca-miR-3958-3p**	0	1	fca-mir-3958
**fca-miR-3958-5p**	0	1	fca-mir-3958
**fca-miR-506-3p**	0	1	fca-mir-506
**fca-miR-508-3p**	0	1	fca-mir-508
**fca-miR-chrD4_30107-3p**	0	1	fca-mir-chrD4_30107
**fca-miR-101b-5p**	1	1	fca-mir-101b
**fca-miR-493-3p**	1	1	fca-mir-493
**fca-miR-7a-2-3p**	1	1	fca-mir-7-2
**fca-miR-487b-3p**	3	1	fca-mir-487b
**fca-miR-186-3p**	4	1	fca-mir-186
**fca-miR-134-5p**	10	1	fca-mir-134
**fca-miR-382-3p**	16	1	fca-mir-382
**fca-miR-126-5p**	17	1	fca-mir-126
**fca-miR-1-2-5p**	0	0	fca-mir-1-2
**fca-miR-106a-3p**	0	0	fca-mir-106a
**fca-miR-1185-5p**	0	0	fca-mir-1185
**fca-miR-1251-3p**	0	0	fca-mir-1251
**fca-miR-1251-5p**	0	0	fca-mir-1251
**fca-miR-128-2-5p**	0	0	fca-mir-128-2
**fca-miR-134-3p**	0	0	fca-mir-134
**fca-miR-138-2-3p**	0	0	fca-mir-138-2
**fca-miR-144-3p**	0	0	fca-mir-144
**fca-miR-150-3p**	0	0	fca-mir-150
**fca-miR-153-3p**	0	0	fca-mir-153-1
**fca-miR-153-3p**	0	0	fca-mir-153-2
**fca-miR-18b-3p**	0	0	fca-mir-18b
**fca-miR-190a-3p**	0	0	fca-mir-190a
**fca-miR-1911-5p**	0	0	fca-mir-1911
**fca-miR-200c-5p**	0	0	fca-mir-200c
**fca-miR-202-3p**	0	0	fca-mir-202

**fca-miR-204-3p**	0	0	fca-mir-204
**fca-miR-205-3p**	0	0	fca-mir-205
**fca-miR-206-5p**	0	0	fca-mir-206
**fca-miR-208b-3p**	0	0	fca-mir-208b
**fca-miR-20a-3p**	0	0	fca-mir-20a
**fca-miR-216a-3p**	0	0	fca-mir-216a
**fca-miR-296-5p**	0	0	fca-mir-296
**fca-miR-299a-1-3p**	0	0	fca-mir-299a-1
**fca-miR-302b-3p**	0	0	fca-mir-302b
**fca-miR-31-3p**	0	0	fca-mir-31
**fca-miR-323a-5p**	0	0	fca-mir-323a
**fca-miR-323b-3p**	0	0	fca-mir-323b
**fca-miR-325-3p**	0	0	fca-mir-325
**fca-miR-325-5p**	0	0	fca-mir-325
**fca-miR-329-5p**	0	0	fca-mir-329
**fca-miR-337-3p**	0	0	fca-mir-337
**fca-miR-337-5p**	0	0	fca-mir-337
**fca-miR-34c-3p**	0	0	fca-mir-34c
**fca-miR-363-3p**	0	0	fca-mir-363
**fca-miR-370-5p**	0	0	fca-mir-370
**fca-miR-375-5p**	0	0	fca-mir-375
**fca-miR-376a-3p**	0	0	fca-mir-376a-1
**fca-miR-376a-3p**	0	0	fca-mir-376a-2
**fca-miR-376a-2-5p**	0	0	fca-mir-376a-2
**fca-miR-376a-3p**	0	0	fca-mir-376a-3
**fca-miR-376b-3p**	0	0	fca-mir-376bc
**fca-miR-376c-3p**	0	0	fca-mir-376c
**fca-miR-376c-5p**	0	0	fca-mir-376c
**fca-miR-380-3p**	0	0	fca-mir-380
**fca-miR-383-3p**	0	0	fca-mir-383
**fca-miR-3959-3p**	0	0	fca-mir-3959
**fca-miR-410-3p**	0	0	fca-mir-410
**fca-miR-411-3p**	0	0	fca-mir-411
**fca-miR-432-3p**	0	0	fca-mir-432
**fca-miR-433-5p**	0	0	fca-mir-433
**fca-miR-449-3p**	0	0	fca-mir-449
**fca-miR-485-3p**	0	0	fca-mir-485
**fca-miR-487a-5p**	0	0	fca-mir-487a
**fca-miR-487b-5p**	0	0	fca-mir-487b
**fca-miR-495-5p**	0	0	fca-mir-495
**fca-miR-497-3p**	0	0	fca-mir-497
**fca-miR-506-5p**	0	0	fca-mir-506
**fca-miR-507a-3p**	0	0	fca-mir-507a
**fca-miR-507b-3p**	0	0	fca-mir-507b
**fca-miR-508-5p**	0	0	fca-mir-508
**fca-miR-514-3p**	0	0	fca-mir-514
**fca-miR-543-5p**	0	0	fca-mir-543
**fca-miR-551b-3p**	0	0	fca-mir-551b
**fca-miR-655-3p**	0	0	fca-mir-655
**fca-miR-656-3p**	0	0	fca-mir-656
**fca-miR-656-5p**	0	0	fca-mir-656
**fca-miR-6715a-5p**	0	0	fca-mir-6715a
**fca-miR-7a-3-3p**	0	0	fca-mir-7-3
**fca-miR-758-3p**	0	0	fca-mir-758

**fca-miR-758-5p**	0	0	fca-mir-758
**fca-miR-802-3p**	0	0	fca-mir-802
**fca-miR-802-5p**	0	0	fca-mir-802
**fca-miR-885-3p**	0	0	fca-mir-885
**fca-miR-889-5p**	0	0	fca-mir-889
**fca-miR-8908n-5p**	0	0	fca-mir-8908n
**fca-miR-9851-3p**	0	0	fca-mir-9851
**fca-miR-9851-5p**	0	0	fca-mir-9851
**fca-miR-99a-3p**	0	0	fca-mir-99a
**fca-miR-chrA2_6163-3p**	0	0	fca-mir-chrA2_6163
**fca-miR-chrA2_6163-5p**	0	0	fca-mir-chrA2_6163
**fca-miR-chrB4_16538-5p**	0	0	fca-mir-chrB4_16538
**fca-miR-chrC2_23695-5p**	0	0	fca-mir-chrC2_23695
**fca-miR-chrE3_33897-3p**	0	0	fca-mir-chrE3_33897
**fca-miR-chrE3_34060-3p**	0	0	fca-mir-chrE3_34060
**fca-miR-chrE3_34323-5p**	0	0	fca-mir-chrE3_34323
**fca-miR-chrX_38640-5p**	0	0	fca-mir-chrX_38640
**fca-miR-chrX_38642-3p**	0	0	fca-mir-chrX_38642
**fca-miR-chrX_38642-5p**	0	0	fca-mir-chrX_38642
**fca-miR-105-5p**	12	0	fca-mir-105
**fca-miR-654-3p**	6	0	fca-mir-654
**fca-miR-31-5p**	4	0	fca-mir-31
**fca-miR-433-3p**	4	0	fca-mir-433
**fca-miR-15a-3p**	3	0	fca-mir-15a
**fca-miR-299a-5p**	3	0	fca-mir-299a
**fca-miR-1185-3p**	2	0	fca-mir-1185
**fca-miR-16-1-3p**	2	0	fca-mir-16-1
**fca-miR-18b-5p**	2	0	fca-mir-18b
**fca-miR-299a-1-5p**	2	0	fca-mir-299a-1
**fca-miR-370-3p**	2	0	fca-mir-370
**fca-miR-381-3p**	2	0	fca-mir-381
**fca-miR-chrA3_6354-5p**	2	0	fca-mir-chrA3_6354
**fca-miR-20b-5p**	1	0	fca-mir-20b
**fca-miR-329-3p**	1	0	fca-mir-329
**fca-miR-371-3p**	1	0	fca-mir-371
**fca-miR-377-5p**	1	0	fca-mir-377
**fca-miR-380-5p**	1	0	fca-mir-380
**fca-miR-487a-3p**	1	0	fca-mir-487a
**fca-miR-885-5p**	1	0	fca-mir-885
**fca-miR-889-3p**	1	0	fca-mir-889

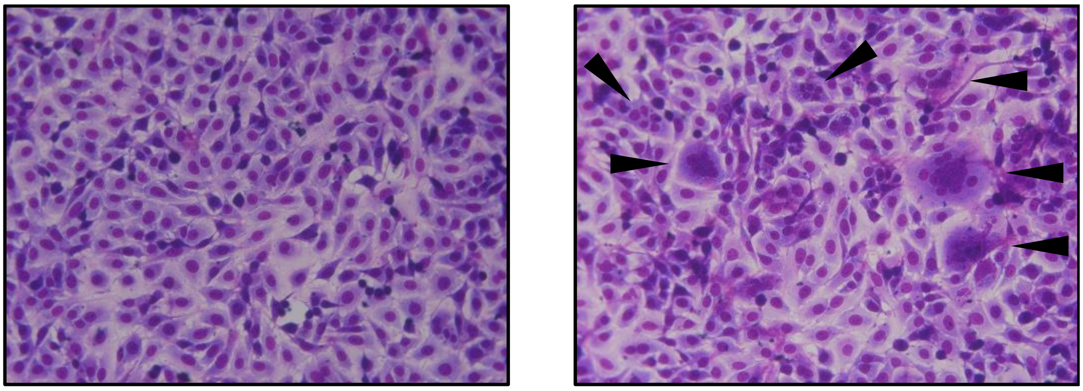

CRFK CRFK/FFV isolate 159

Fig. S1. CPE induced by FFV isolate 159.
